# Identification of a Seed Vigor–Related QTL Cluster Associated with Weed Competitive Ability in Direct–Seeded Rice (*Oryza Sativa* L.)

**DOI:** 10.1186/s12284-023-00664-x

**Published:** 2023-10-13

**Authors:** Shan Xu, Yuexin Fei, Yue Wang, Wenjia Zhao, Luyan Hou, Yujie Cao, Min Wu, Hongkai Wu

**Affiliations:** College of Advanced Agricultural Sciences, Zhejiang A & F University, Hangzhou, 311300 Zhejiang China

**Keywords:** Direct–seeded rice (*Oryza sativa* L.), Seed vigor, Seedling vigor, Early vigor, Seedling stand density (SSD), Weed competitive ability (WCA), Quantitative trait locus (QTL) cluster

## Abstract

Direct seeding of rice (*Oryza sativa* L.) is a low-labor and sustainable cultivation method that is used worldwide. Seed vigor and early vigor are important traits associated with seedling stand density (SSD) and weed competitive ability (WCA), which are key factors in direct-seeded rice (DSR) cultivation systems. Here, we developed a set of chromosome segment substitution lines with Xiushui134 as receptor parent and Yangdao6 as donor parent and used these lines as a mapping population to identify quantitative trait loci (QTLs) for seed vigor, which we evaluated based on germinability–related indicators (germination percentage (GP), germination energy (GE), and germination index (GI)) and seedling vigor–related indicators (root number (RN), root length (RL), and shoot length (SL) at 14 days after imbibition) under controlled conditions in an incubator. Ten QTLs were detected across four chromosomes, of which a cluster of QTLs (*qGP11*, *qGE11*, *qGI11*, and *qRL11*) co-localized on Chr. 11 with high LOD values (12.03, 8.13, 7.14, and 8.75, respectively). Fine mapping narrowed down the QTL cluster to a 0.7-Mb interval between RM26797 and RM6680. Further analysis showed that the QTL cluster has a significant effect (*p* < 0.01) on early vigor under hydroponic culture (root length, total dry weight) and direct seeding conditions (tiller number, aboveground dry weight). Thus, our combined analysis revealed that the QTL cluster influenced both seed vigor and early vigor. Identifying favorable alleles at this QTL cluster could facilitate the improvement of SSD and WCA, thereby addressing both major factors in DSR cultivation systems.

**Supplementary Information:**

The online version contains supplementary material available at 10.1186/s12284-023-00664-x.

## Background

Rice (*Oryza sativa* L.), an important global food crop, is traditionally cultivated by transplanting seedlings into puddled soil (Kumar and Ladha 2011). However, puddling and transplanting consume 30% of the total water requirements of rice, and require much energy and labor (Raj and Syriac 2017). Various problems, such as labor scarcity due to a shift of rural laborers to the city, are increasing production costs and reducing profits to farmers (Mondal et al. 2020; Raj and Syriac 2017), making rice production via transplantation increasingly unsustainable (Farooq et al. 2011; Mondal et al. 2020). Direct seeding is a good alternative to transplantation, with advantages that include lower labor requirements, less need for water and energy (Liu et al. 2014; Sun et al. 2015), and increased profitability to farmers (Mondal et al. 2020). More importantly, direct–seeded rice (DSR) matures 7 to 10 days earlier than conventional transplanted rice (TPR) due to the absence of transplant shock and faster seedling establishment (Raj and Syriac 2017; Rana et al. 2014) while being almost on par with TPR in terms of yield (Awan, Manzoor, Ali, Safdar, & Yaqub, 2007). Therefore, in recent years, there has been a shift from TPR to DSR cultivation in several Southeast Asian countries (Sun et al. 2015). Studies in countries including China, India, Sri Lanka, Nepal, and Australia indicated that rice can be successfully cultivated using direct seeding (Mondal et al. 2020). In some parts of Southeast Asia (such as the Philippines, Malaysia, and Vietnam), transplantation is widely being replaced by wet direct seeding in puddled soils (Mondal et al. 2020).

Despite its advantages over TPR, DSR is subject to two major biological constraints (Raj and Syriac 2017). The first is non–uniform seedling stand density (SSD, seedling emergence performance). In TPR cultivation, seeds germinate and seedlings are grown under controlled conditions in nurseries, whereas in DSR cultivation, the seeds and seedlings are exposed to adverse environmental conditions during germination and emergence, which can lead to non–uniform SSD (Qi et al. 2012). To ensure uniform SSD, a high sowing rate is usually recommended (Sun et al. 2015), but this wastes seeds and results in high population density and low quality. The second major constraint is high weed infestation (poor weed control) (Chauhan and Abugho 2013; Kumar and Ladha 2011; Singh et al. 2017). Under direct seeding, the emergence of weeds concurrently with rice seedlings and the wide adaptability and competitive advantage of weeds can make weeds dominate the crop habitat, which adversely affects crop yields (Raj and Syriac 2017).

The key to obtaining a uniform SSD is to use seeds that exhibit high performance during germination and seedling emergence, especially during the growth of the first leaf, when adventitious roots normally emerge in a timely manner. These seed properties, referred to collectively as seed vigor, determine seed germinability and seedling vigor (Perry 1978). Seed vigor is thus an essential trait affecting SSD in direct seeding systems.

The weed competitive ability (WCA) of crops is closely linked to several traits, including plant height, tiller number, early biomass accumulation, shoot and root dry weight, leaf area index and specific leaf area during vegetative growth, and canopy ground cover (Caton et al. 2003; Dimaano et al. 2020; Dingkuhn et al. 1999; Fischer et al. 1997; Lu et al. 2007; Mahajan et al. 2014; Okami et al. 2011; Raj and Syriac 2017; Zhao et al. 2006). All these characteristics are expressed as early vigor (i.e., the ability of a plant to grow relatively rapidly during the early growth stages), which is an important trait associated with the ability of a crop to suppress weeds.

However, improving seed vigor and early vigor via conventional breeding is difficult due to the quantitative inheritance of these two traits (Redoña and Mackill 1996; Singh et al. 2017). Marker-assisted selection (MAS) can improve breeding efficiency through the exploitation and introgression of favorable alleles into commercial varieties. This process requires the identification of genes/quantitative trait loci (QTLs) for seed vigor and early vigor.

Many efforts have been made to identify QTLs and regulatory genes for seed vigor (Zhao et al. 2021), as well as QTLs for early vigor or strong WCA (Bharamappanavara et al. 2020; Cairns et al. 2009; Cui et al. 2002, 2007; Redoña and Mackill 1996; Singh et al. 2017; Thapa and Septiningsih 2021; Xie et al. 2014; Xu et al. 2004; Z.-H. Zhang et al. 2005; Zhang 2004; Zhou et al. 2007). However, to date, no studies combined the analysis of QTLs for seed vigor (which governs SSD) and for early vigor (which favors weed suppression) to link the genetic architectures of seed vigor and early vigor. Here, we identified candidate QTLs associated with seed vigor under controlled conditions and investigated their effects on early vigor in rice under hydroponic culture and direct seeding conditions. This combined analysis helped us identify additional common QTLs for both seed vigor and early vigor. These QTLs could both improve uniform SSD and enhance WCA in DSR cultivation systems and thereby increase the feasibility and uptake of DSR cultivation.

## Materials and Methods

### Plant Materials

**Fig. 1:**
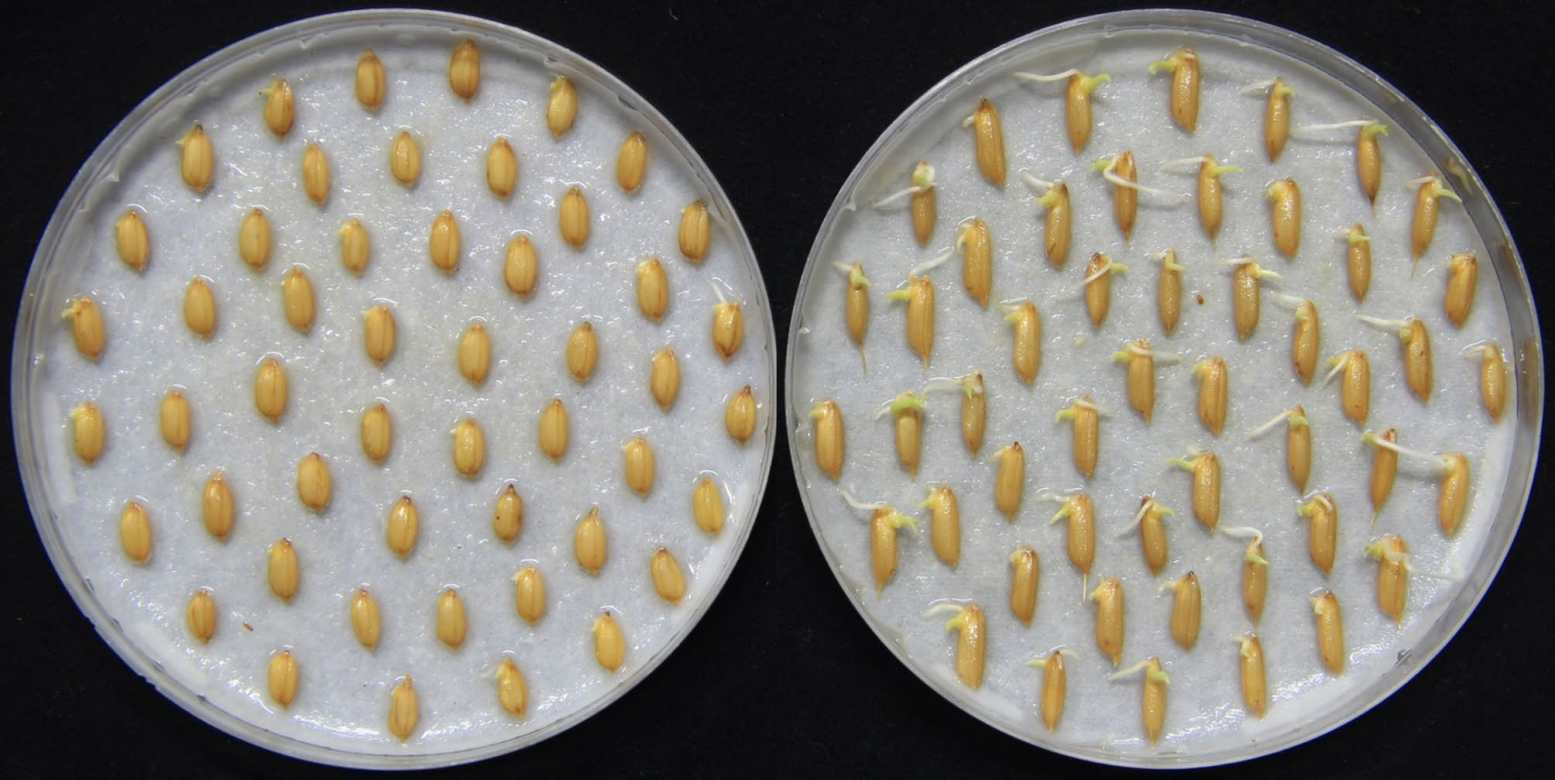
Germination performance of Xs134 (**left**) and Yd6 (**right**) at 48 h after imbibition

**Fig. 2:**
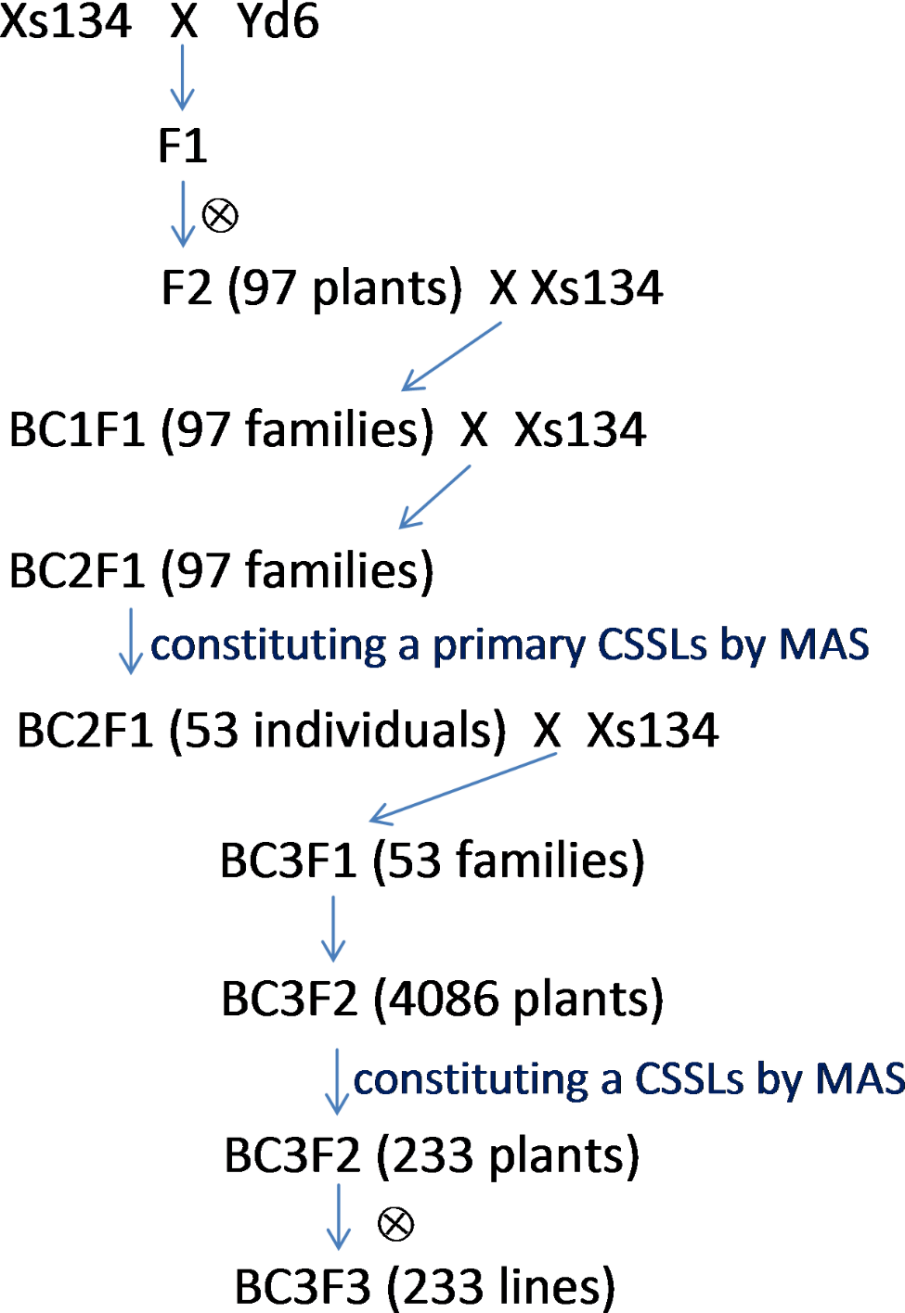
Schematic diagram of the strategy used to develop the CSSLs

A set of chromosome segment substitution lines (CSSLs) with Xiushui134 (Xs134) as the receptor parent and Yangdao6 (Yd6) as the donor parent was developed and used in this study. Xs134, a *japonica* cultivar, is grown extensively in south China but has poor seed germinability (Fig. 1). Yd6 is an *indica* super parent with strong seed germinability that has been used to produce many hybrid varieties, such as the super hybrid rice cultivars LYP9 and YLY1, which are commercially cultivated widely in China. Figure 2 shows a schematic diagram of the strategy used to develop the CSSLs. The F1 seeds were generated by crossing Xs134 with Yd6 and was then advanced to the F2 generation by selfing. In the F2 population, 97 individuals were randomly selected as the female parents and backcrossed with Xs134 to generate 97 BC1F1 families. One individual from each BC1F1 family was randomly selected and backcrossed (as female parent) with Xs134 to generate 97 BC2F1 families.

A set of 12 randomly selected individuals from each BC2F1 family was genotyped in the first round using a set of polymorphic markers (71 SSRs and 24 insertions/deletions); information about these markers is shown in Table S1, and a linkage map is shown in Fig. S1. The 53 resulting individuals (founders, with a known genotype) constituted a set of primary CSSLs, with each individual containing one or a few overlapping marker-defined heterozygous segments that together covered the entire donor genome. All 53 individuals were used as females in backcrosses with Xs134, generating 53 BC3F1 families. Approximately 96 plants per BC3F1 family were generated, yielding a total of 4086 BC3F2 plants, each of which can be traced to a founder (one of 53 individuals). All these plants were screened by MAS for various backcross progenies with targeted introgressed donor chromosome segments based on their genotypic content using GGT (van Berloo 2008), a graphical genotyping software program, to generate a set of 233 CSSLs (Fig. S2). Each line contained one or a few homozygous donor segments, which together covered the entire donor genome. Each marker locus had ≥ 3 replicates of donor segments, with each replicate corresponding to a CSSL (Fig. S2).

The CSSLs were grown during the regular growing season in 2017 at the Experiment Station of Zhejiang Agricultural and Forestry University, Linan, China (30.16 °N, 120.12 °E). On the about 45 days after heading when seeds turned yellow, the seeds were harvested, air-dried in ambient conditions to ~13% moisture, stored at room temperature (~25°C) for three months for after-ripening and afterward in the cold room (16 °C with < 65% relative humidity) until being used to phenotype for seed vigor.

### Phenotyping the CSSLs for Seed Vigor

Seed vigor determines seed germinability and seedling vigor. Seed germinability was evaluated using a germination test in which 100 seeds were placed in a germination box (12 cm × 12 cm × 6 cm) containing wet foam paper (in three replications) in an incubator with day/night temperatures of 30/20^◦^C and a 13-h/11-h day/night cycle. Cumulative germination count was recorded daily from 2 to 14 days after imbibition (DAI) when the radicle length of a germinated seed was approximately equal to the seed length and the coleoptile length was approximately half the seed length. Seed germinability is reflected by the germination percentage (GP), germination energy (GE), and germination index (GI). Seedling vigor is reflected by the root number plant^−1^ (RN), root length (RL), and shoot length (SL), which were determined using the same germination test as described above, except that 50 seeds were placed in a germination box, and the average values are based on 12 seedlings sampled from each replicate at 14 DAI. These traits are described in Table 1.

**Table 1:** Descriptions of traits related to seed vigor and early vigor investigated in the study

Traits	Observations	Descriptions
*Seed vigor measured under controlled condition in an incubator*		
Germination percentage	GP	The percentage of germinated seeds at 14 DAI
Germination energy	GE	The percentage of germinated seeds at 5 DAI
Germination index	GI	∑(Gt/Dt), where Gt is number of germinated seed on Day t.
Root number	RN	Number of root plant^− 1^ at 14 DAI
Root length	RL	Max. length of root at 14 DAI
Shoot length	SL	Max. length of shoot at 14 DAI
*Early vigor estimated under hydroponic culture condition in the green house*		
Root number	RN_21_	Number of root plant^− 1^ at 21 DAI
Root length	RL_21_	Max. length of root at 21 DAI
Plant height	PH_21_	Plant height at 21 DAI
Total dry weight	TDW_21_	Total (shoot + root) dry weight plant^− 1^ at 21 DAI
*Early vigor estimated under direct seeding condition in the field*		
Plant height	PH_28_	Plant height at 28 DAS
Tiller number	TN_28_	Number of tiller hill^− 1^ at 28 DAS
Aboveground dry weight	ADW_28_	Aboveground dry weight hill^− 1^ at 28 DAS

Abbreviations: DAI, days after imbibition; DAS, days after seeding

### Analysis of QTLs for Seed Vigor

QTL analysis was performed by the RSTEP-LRT-ADD (Stepwise regression based-likelihood ratio tests for additive QTL) method for the CSL functionality provided by the QTL IciMapping Version 4.1 software package (Meng et al. 2015), which was well suited for the CSSLs (Alam et al. 2011). Threshold value of condition number for reducing multi-collinearity among marker variables was specified as -1, which means that only duplicate markers will be deleted before QTL mapping. PIN (probability in stepwise regression) was set to 0.01, which means that the largest P-value for entering variables in stepwise regression of phenotype on marker variables is 0.01, and the largest P-value for removing variables is 0.02. A standard threshold logarithm of odds (LOD) score of 3.0 was used to suggest the presence of a putative QTL.

### Validating and Fine Mapping the Putative QTL Cluster

**Fig. 3:**
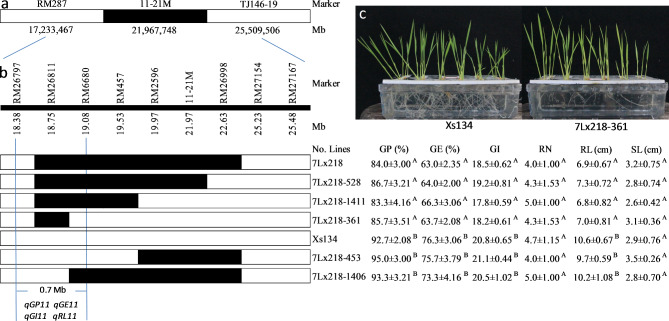
Fine mapping of the QTL cluster for seed vigor on Chr. 11. **a**, The QTL cluster region targeted in substituted donor segment of the CSSL 7Lx218. **b**, Substitution mapping of the QTL cluster, showing genotype and phenotype of the NILs composed of 5 recombinants along with 7Lx218 and Xs134 (receptor parent). Seed germinability (GP, GE, and GI) and seedling vigor (RL, SL, and RN at 14 DAI) are expressed as means ± standard deviation (SD). The different superscript letters (A and B) mean that their genotypic means significantly differ at *p* < 0.01; the same superscript letters (A and A) mean that their genotypic means have no significant difference at *p* > 0.1. **c**, Morphological characteristics of the NIL 7Lx218-361 and Xs134 under hydroponic culture condition at 21 DAI.

A cluster of QTLs (*qGP11*, *qGE11*, *qGI11*, and *qRL11*) was detected near marker 11-21 M on Chr. 11. The CSSL 7Lx218 harbored only one marker (11-21 M)-defined donor segment (Table S2; Fig. S2; Fig. 3a). To validate the putative QTL cluster, 7Lx218 and Xs134 were grown in 2018 as described above, and their seed vigor was compared. Furthermore, 7Lx218 was crossed with Xs134 to produce a sub-F2 population, and SSR markers were selected from the Rice Universal Core Genetic Map (Orjuela et al. 2009) and the list provided by IRGSP (Sasaki 2005) to identify polymorphisms between Xs134 and Yd6 at the interval flanked by RM287 and TJ146-19. These polymorphic markers were used for fine mapping of the QTL cluster by substitution mapping (Paterson et al. 1990; Tan et al. 2020; Wissuwa et al. 2002). During the fine mapping, a set of near-isogenic lines (NILs) of the QTL cluster was obtained (Fig. 3b).

### Studying the Effects of the QTL Cluster on Early Vigor

The NIL 7Lx218-361 harboring the QTL cluster, together with Xs134 as a control, was used to conduct a hydroponic culture experiment in the greenhouse to study the effects of the QTL cluster on early vigor. Before the experiment, 50 seeds per sample were germinated at 25^◦^C. The germinated seeds were sown sequentially based on the order of germination in a cell tray (cell count: 6 × 6; tray size: 15 cm × 30 cm; depth of cell: 1 cm) (Fig. 3c), and the trays were placed in a greenhouse where the temperature was controlled at 25^◦^C under natural light conditions. The plants were subjected to hydroponic culture conditions as described by (Yoshida et al. 1976). Estimations of early vigor, including RN_21_, RL_21_, PH_21_, and TDW_21_ (Table 1), were made based on average values of 12 plants selected from the middle four columns of each replicate at 21 DAI. TDW_21_ of the samples was calculated after oven drying at 105^◦^C for 0.5 h, closely followed by 80^◦^C for 72 h.

The field experiment was conducted by direct seeding using a completely randomized design in six replications with a plot size of 1 × 2 m^2^ (14 rows × 4 columns). Uniformly germinated seeds were sown at a soil depth of 1 cm in furrows, and the furrows were covered by vegetable garden soil. The experimental field was irrigated based on soil moisture levels, which were determined by recording the water level (−3 cm) of the ditch. All weeds were removed by manual weeding during the cropping period. Compound fertilizer with 25% (12% N + 7% P_2_O_5_ + 6% K_2_O) nutrient content was applied at a rate of 375 kg ha^−1^ at the time of sowing. Early vigor, including PH_28_, TN_28_, and ADW_28_ (Table 1), was estimated as described above, except that sampling was performed at 28 days after sowing (DAS).

### Data Analysis

Analysis of variance (ANOVA) was carried out to compare the genotypic means of seed vigor and early vigor using the least significance difference (LSD) test. The percentage data for GP and GE were arcsine square root transformed before being subjected to ANOVA. Data analysis was performed using SPSS Statistics version 17.0 software for Windows (SPSS Inc., Chicago, IL, USA).

## Results

### Variation in Seed Vigor in the CSSLs

**Fig. 4:**
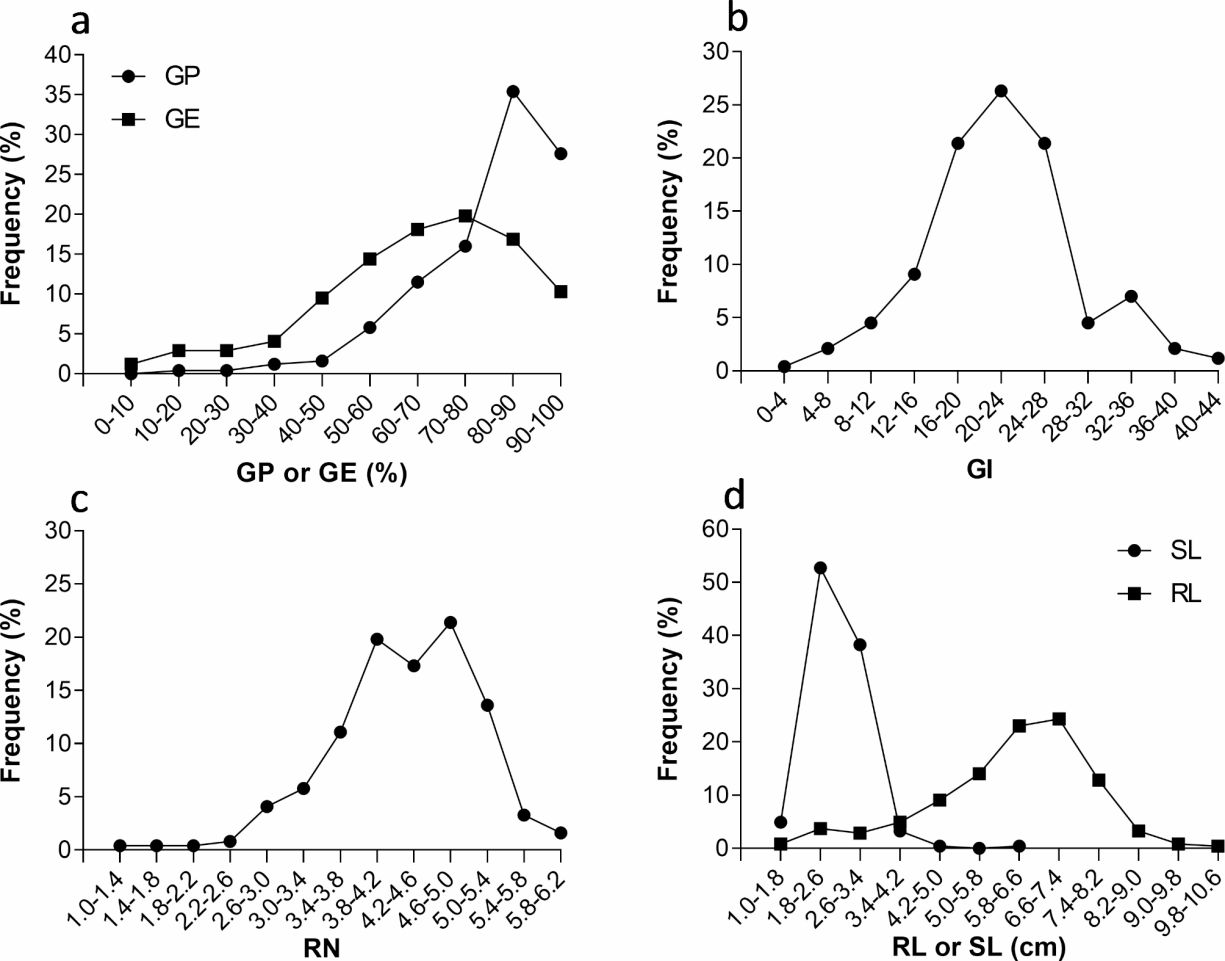
Phenotypic distribution for seed vigor–related indicators, GP and GE (**a**), GI (**b**), RN (**c**), and SH and RL (**d**), in the CSSLs

We investigated six seed vigor–related indicators, including seed germinability (GP, GE, and GI) and seedling vigor (RL, SL, and RN at 14 DAI), in the CSSL population (Table 2) and constructed biplots with the indicators in rows and frequency in columns (Fig. 4). The classical quantitative genetics hypothesizes that variation in a specific quantitative trait in a segregating population follows a normal distribution then this trait may be controlled by many genes, each with a small effect. In this study, all six indicators exhibited continuous distributions in the population, instead of following a normal distribution, indicating that a large amount of genetic variation and some major QTLs existed in the population. Transgressive segregants were observed for the six indicators. Some segregants performed better and others performed worse than the receptor parent (Xs134) (Table 2), suggesting that each indicator is controlled by multiple genes and that some alleles from the donor parent have positive effects while others have negative effects.

**Table 2:** Descriptive statistics of the seed vigor traits evaluated in the CSSLs

Traits	Receptor parent	CSSLs			
		Peak value interval	Mean	Range	SD
GP (%)	90	80–90	80.25	18–99	14.45
GE (%)	71	70–80	65.65	4–97	20.54
GI	24.31	20–24; 32–36	21.98	2.77–43.43	6.92
RN	4.4	3.8–4.2; 4.6-5.0	4.26	1.2–5.9	0.79
RL (cm)	7.89	6.6–7.4	6.06	1.55–10.27	1.57
SL (cm)	3.12	1.8–2.6	2.56	1.4–5.96	0.51

Abbreviations: SD, standard deviation; the others are described in Table 1

### Correlation Analysis Among Seed Vigor–Related Indicators

GP, GE, and GI values, derived from a set of relational germination data, are widely used to estimate seed germinability. Significant correlations existed among GP, GE, and GI (Table 3). The highest correlation coefficient (0.921) was detected between GE and GI. We detected significant positive correlations (*p* < 0.01) between seed germinability (GP, GE, and GI) and seedling vigor (RL, SL, and RN at 14 DAI), suggesting that seed germinability may translate into seedling vigor.

**Table 3:** Correlations among the seed vigor traits evaluated in the CSSLs

	GP	GE	GI	RL	SL	RN
GP	1	0.813^**^	0.799^**^	0.330^**^	0.502^**^	0.445^**^
GE	0.813^**^	1	0.921^**^	0.446^**^	0.578^**^	0.502^**^
GI	0.799^**^	0.921^**^	1	0.489^**^	0.579^**^	0.482^**^
RL	0.330^**^	0.446^**^	0.489^**^	1	0.460^**^	0.521^**^
SL	0.502^**^	0.578^**^	0.579^**^	0.460^**^	1	0.517^**^
RN	0.445^**^	0.502^**^	0.482^**^	0.521^**^	0.517^**^	1

** *p* < 0.01

### Mapping and Validation of QTLs for Seed Vigor

We identified 10 QTLs at 6 loci across 4 chromosomes (Table 4). Only one QTL each (*qSL3*, *qRL7*, *qGP8*, and *qRN11*) was detected at loci 1, 3, 4, and 6, respectively, with respective LOD scores of 4.96, 3.75, 4.11, and 7.34. Positive alleles of *qSL3* and *qRL7* were derived from Yd6, and positive alleles of *qGP8* and *qRN11* were derived from Xs134. The phenotypic variation explained (PVE) by *qSL3*, *qRL7*, *qGP8*, and *qRN11* was 8.97%, 6.51%, 6.27%, and 11.73%, respectively. Two QTLs (*qGE7* and *qGI7*) co-localized at locus 2 on Chr. 7, with low LOD values (2.97 and 5.32, respectively). Nonetheless, there was a highly significant positive correlation coefficient of 0.921 between GE and GI (Table 3), suggesting that *qGE7* and *qGI7* are the same locus or closely linked loci.

**Table 4:** QTL analysis of seed vigor in the CSSLs

Locus	Chr.	Nearest marker	Position (Mb)	QTL	LOD	Add	PVE (%)	Donor of positive allele
1	3	RM570	35.6	*qSL3*	4.96	0.42	8.97	Yd6
2	7	7-4.5 M	4.5	*qGE7*	2.97	5.86	5.16	Yd6
				*qGI7*	5.32	4.03	9.19	Yd6
3	7	RM1132	24.0	*qRL7*	3.75	1.13	6.51	Yd6
4	8	RM5545	28.3	*qGP8*	4.11	-6.13	6.27	Xs134
5	11	11-21 M	22.0	*qGP11*	12.03	-11.46	19.04	Xs134
				*qGE11*	8.13	-12.45	14.75	Xs134
				*qGI11*	7.14	-5.88	12.43	Xs134
				*qRL11*	8.75	-1.50	16.04	Xs134
6	11	RM6094	28.9	*qRN11*	7.34	-1.08	11.73	Xs134

Add that denotes additive effect is the effect of substituting a Yd6 allele for corresponding Xs134 allele; its positive value indicates that Yd6 has the positive allele; the case of negative values is just the opposite

A cluster of QTLs (*qGP11*, *qGE11*, *qGI11*, and *qRL11*) co-localized at locus 5 on Chr. 11, with high LOD values (12.03, 8.13, 7.14, and 8.75) and high PVE (19.04, 14.75, 12.43, and 16.04), respectively, and negative additive effects of alleles from the donor. To validate the results, we identified the CSSL 7Lx218, containing only one substituted donor segment targeting the QTL cluster at an interval between markers RM287 and TJ146-19 (Table S2; Fig. S2; Fig. 3a). We grew this line together with Xs134 and compared the values for the six indicators at 14 DAI between 7Lx218 and Xs134 (Table 5). The GP, GE, GI, and RL were significantly lower for 7Lx218 than for Xs134 (*p* < 0.01), while there was no significant difference in SL or RN; these results coincided with the mapping results described above, which confirmed the presence of the QTL cluster.

**Table 5:** Comparisons of seed vigor–related indicators at 14 DAI between the CSSL 7Lx218 and Xs134

Lines	GP (%)	GE (%)	GI	RN	RL (cm)	SL (cm)
7Lx218	78 ± 3.37**	62 ± 3.06**	17.40 ± 0.86**	4.4 ± 0.52^NS^	5.07 ± 0.43**	2.80 ± 0.27^NS^
Xs134	91 ± 4.64 ^a^	73 ± 3.89	23.99 ± 1.02	4.3 ± 0.48	7.32 ± 0.43	3.43 ± 0.23

^a^ Data presented are mean values with standard deviation (SD)

** *p* < 0.01; ^NS^ not significant at *p* > 0.1

### Fine Mapping of the QTL Cluster for Seed Vigor

To uncover the gene(s) underlying this QTL cluster, we performed substitution mapping to determine the precise location of each QTL. We assessed 23 SSR markers for the substituted interval of 7Lx218 between RM287 and TJ146-19. Nine of these markers (RM26797, RM26811, RM6680, RM457, RM2596, 11-21 M, RM26998, RM27154, and RM27167) detected differences between Yd6 and Xs134, and six of the nine (RM26811, RM6680, RM457, RM2596, 11-21 M, and RM26998) detected polymorphisms between 7Lx218 and Xs134 (Fig. 3b). These results indicated that 7Lx218 carried a substituted segment between markers RM26797 and RM27154, with an estimated length of 6.85 Mb, based on the Nipponbare Reference Genome (IRGSP_1.0).

We crossed 7Lx218 with Xs134 to develop a sub-F2 population. We genotyped 1537 individuals from this population at the six polymorphic marker loci and obtained five homozygous recombinants. These recombinants, along with 7Lx218 and Xs134, composed our set of NILs (Fig. 3b). We phenotyped these NILs for seed vigor (GP, GE, GI, RN, RL, and SL), finding that the NILs fell into two phenotypic classes. In class 1, comprising 7Lx218-453, 7Lx218-1406, and Xs134 (receptor parent), no significant difference was detected for all six indicators, indicating that they contained no QTLs in the substituted segment defined by RM26811 and RM27154. In class 2, comprising 7Lx218-528, 7Lx218-1411, 7Lx218-361, and 7Lx218, GP, GE, GI, and RL were significantly lower than the values for class 1 (*p* < 0.01), although there was no significant difference in SL or RN between class 1 and class 2, and their substitution segments overlapped between markers RM26797 and RM6680. Based on these results, we narrowed down the QTL cluster (*qGP11*, *qGE11*, *qGI11*, and *qRL11*) to an estimated interval of 0.7 Mb between RM26797 and RM6680, with negative effects of the allele from the donor at the QTL cluster on GP, GE, GI, and RL (Fig. 3b).

### Studying the Effects of the QTL Cluster on Early Vigor

Early seed germination and early seedling growth are crucial for early vigor parameters such as plant height, number of tillers, shoot fresh weight, and shoot dry weight (Dimaano et al. 2020); in turn, early vigor is closely linked to WCA. The critical period of weed competition is 14–41 DAS (Bhagirath S. Chauhan and Johnson 2011). The NIL 7Lx218-361 carried a single substituted segment, with an estimated length of 0.7 Mb, targeting the QTL cluster (Fig. 3b). To study the effects of the QTL cluster on early vigor, we performed hydroponic culture experiments in the greenhouse and direct seeding experiments in the field. When we assessed plant grown under hydroponic culture conditions at 21 DAI, we observed only a few differences in shoots, but many differences in roots, between 7Lx218-361 and Xs134 (Fig. 3c). The PH_21_ was higher and the RN_21_ was lower for 7Lx218-361 compared to Xs134, but these differences were not significant (Table 6). However, the RL_21_ and TDW_21_ were significantly lower for 7Lx218-361 than for Xs134 (*p* < 0.01). These results indicated that the QTL cluster had a significant effect on early vigor in plants grown in hydroponic culture for 21 days, with the allele from the donor having a negative effect on this trait. We obtained similar results at 28 DAS under direct seeding conditions in the field (Table 7). Both TN_28_ and ADW_28_ were significantly lower for 7Lx218-361 than for Xs134, though PH_28_ did not significantly differ between 7Lx218-361 and Xs134. These results further confirmed that the QTL cluster was responsible for both seed vigor and early vigor and that the allele from the donor had negative effects on these traits.

**Table 6:** Comparisons of early vigor–related indicators at 21 DAI between the NIL 7Lx218-361 and Xs134 under hydroponic culture conditions in the greenhouse

Lines	RN_21_	RL_21_ (cm)	PH_21_ (cm)	TDW_21_ (g)
7LX218-361	7.33 ± 1.03^NS^	8.87 ± 3.23**	9.98 ± 0.84 ^NS^	0.0173 ± 0.0018*
Xs134	7.50 ± 0.55 ^a^	15.65 ± 3.97	9.07 ± 0.72	0.0192 ± 0.0028

^a^ Data presented are mean values with standard deviation (SD)

* *p* < 0.05; ** *p* < 0.01; ^NS^ not significant at *p* > 0.1

**Table 7:** Comparisons of early vigor–related indicators at 28 DAS between the NIL 7Lx218-361 and Xs134 under direct seeding conditions in the field

Lines	TN_28_	PH_28_ (cm)	ADW_28_ (g)
7LX218-361	5.3 ± 0.42**	17.6 ± 0.32^NS^	0.37 ± 0.029**
Xs134	6.7 ± 0.56 ^a^	18.5 ± 0.41	0.49 ± 0.036

^a^ Data presented are mean values with standard deviation (SD)

** *p* < 0.01; ^NS^ not significant at *p* > 0.1

## Discussion

Direct-seeded rice is increasingly being cultivated in many Asian countries due to the lower labor requirements and operational simplicity compared to transplanted rice. Rice varieties suitable for direct seeding must possess two major characteristics: high seed vigor and high early vigor. Seed vigor is the ability of germinated seeds to develop roots and shoots and grow into seedlings. After the seed reserve is depleted (at ~ 14 DAI), seedlings with roots and shoots enter the physiological transition period from heterotrophic to autotrophic growth. Thus, early plant development involves three stages: germination, heterotrophic growth (seedling growth), and early autotrophic growth (vegetative growth); the performance at these stages is referred to as germinability, seedling vigor, and early vigor, respectively (Lu et al. 2007). As germination and seedling growth rely on seed reserves, seed vigor (i.e., the sum total of a seed’s properties) determines seed germinability and seedling vigor, which govern uniform SSD. Once plants enter autotrophic growth, they rely on nutrients from the soil and photosynthesis to develop tillers, leaf area, and biomass in an exponential growth pattern before canopy closure. This performance, which is also expressed as early vigor, favors weed suppression via early canopy closure (Rebolledo et al. 2012; Singh et al. 2017).

As germination and seedling growth are closely linked to early vegetative growth, seed vigor may be associated with early vigor. In the current study, seed germinability (GP, GE, and GI) was positively correlated with seedling vigor (RL, RN, and SL at 14 DAI) (Table 3). Similarly, previous studies demonstrated that germination rate and seedling vigor are positively correlated with field emergence, seedling establishment, plant height, and seedling dry weight (Dimaano et al. 2017, 2020; Mahajan et al. 2014; Wang et al. 2010; A. Zhang et al., 2017). High seed vigor leads to rapid seed germination and rapid seedling growth, thereby ensuring vigorous seedling stand growth, with rapid canopy development giving rice plants an initial advantage over weeds (Anwar et al. 2012). Thus, seed vigor estimated under controlled conditions is thought to translate into early vigor under field conditions.

Seed vigor is commonly evaluated based on germination-related indicators (GP, GE, and GI) and seedling growth–related indicators (RN, RL, SH, and DW before 14 DAI). Measuring these indicators under controlled conditions (such as in an incubator) provides repeatable data, which should have good observation value. By contrast, early vigor–related indicators (RN, RL, SH, and DW after 14 DAS) are difficult to measure under field conditions due to the influence of climate, soil, and fertility level. Therefore, it is preferable to initially map a QTL for seed vigor in a population under controlled conditions and then validate its effect on early vigor under field conditions. The resulting QTLs should both improve SSD and enhance WCA, thereby addressing both major constraints of DSR cultivation systems.

In the current study, by examining seed vigor under controlled conditions in an incubator, we detected 10 QTLs for seed vigor at 6 loci consisting of three loci with positive effect and three loci with negative effect (Table 4), and identified a QTL cluster for seed vigor consisting of *qGP11*, *qGE11*, *qGI11*, and *qRL11*, which co-localized in a 0.7-Mb genomic region (Fig. 3). As expected, the QTL cluster had a significant effect (*p* < 0.01) on early vigor (RL_21_ and TDW_21_) under hydroponic culture conditions in the greenhouse and on early vigor (TN_28_ and ADW_28_) under direct seeding conditions in the field. Thus, the QTL cluster influenced both seed vigor and early vigor. This QTL cluster was identified under diverse conditions, with high LOD values (12.03, 8.13, 7.14, and 8.75, respectively; Table 4), indicating that this is a reliable QTL cluster.

Unexpectedly, at the major QTL cluster for seed vigor, the positive effect allele isn’t from Yd6 that has stronger germinability than Xs134. Most agronomically important traits are quantitative in practice. QTL mapping experiment can uncover the genetic basis of these traits by determining the number, locations, gene effects of loci involved and their interactions with other loci (epistasis). However, only a small number of these loci are detectable in a mapping population largely because of epistasis (Li 2001) which has been recognized as an important genetic basis underlying complex phenotypes. In addition, it is difficult to detect a minor QTL with a relatively small effect. Using a set of backcross inbred lines derived from the cross between Habataki (nonrecurrent parent with more grain number per panicle) and Koshihikari (recurrent parent with smaller grain number per panicle), only five QTLs for grain number, consisting of two with positive effect and three with negative effect, were detected (Ashikari et al. 2005). Thus, One largely possible reason is that many minor QTLs bearing Yd6 positive main effects were not detected; the other one is that there exist some epistasic QTLs. These QTLs, together, explained phenotypic variance as two important genetic component determining seed vigor.

We compared the newly identified QTL cluster with previously reported QTLs associated with seed vigor, seedling vigor, and early vigor, including WCA traits such as shoot length, shoot weight, coleoptile length, shoot dry weight, leaf area, and specific leaf area, by converting genetic positions into physical positions by Primer-BLAST based on the Nipponbare IRGSP_1.0 Reference (https://rapdb.dna.affrc.go.jp/tools/blast). Two QTLs, *qGP-11* (Chr. 11: 151,66,527–18,874,200 Mb) (Z.-f. Wang, Wang, Bao, Wang, & Zhang, 2010) and *qGE-11* (Chr. 11: 18,768,641 Mb) (Guo et al. 2019), co-localized with the QTL cluster identified in this study (Chr. 11: 18,382,045–19,082,768 Mb). However, each of these previously identified QTLs affected only one of three indicators, GP, GE, and GI, and no reports are available on seedling vigor or early vigor. Therefore, whether the two previously reported QTLs are identical to the QTL cluster identified in this study remains unclear.

Many QTL clusters (also known as QTL hotspots) have been identified on different chromosomes for various co-localized traits (Almeida et al. 2014; Bharamappanavara et al. 2020; Cai and Morishima 2002; Cai et al. 2017; Crowell et al. 2016; Guan et al. 2020; Liu et al. 2013; Mia et al. 2019; Singh et al. 2017; Sivasakthi et al. 2018; Xie et al. 2007). Two major scenarios, pleiotropy (identical locus) and linkage (disparate locus), can account for QTL clusters. Pleiotropy often contributes to positive correlation between traits having physiological (causal) relationships (Li 2001). As germination is closely linked to seedling growth, the QTL cluster arises possibly from pleiotropy, that is to say an identical locus. However, it needs to be validated by disassembling the QTL cluster into one or more loci. Anyway, QTL clusters are ideal for improving rice varieties through marker-assisted breeding because they simultaneously improve several traits, such as seed vigor and early vigor, as demonstrated in this study. Despite the negative effect of the allele from the donor at the QTL cluster on target traits, we delimited the QTL cluster to a 0.7-Mb genomic region, which sets the stage for disassembling the QTL cluster into one or more loci. When one locus underlies a QTL cluster, favorable alleles (haplotypes) can be explored. When multiple linked loci underlie a QTL cluster, favorable haploblocks can be explored by combining favorable alleles for the multiple linked loci from germplasm resources, such as the receptor parent Xs134. These favorable haplotypes or haploblocks at this QTL cluster are under positive selection, which could facilitate the improvement of rice varieties for traits beneficial for direct seeding (Cai et al. 2017).

## Conclusions

Seed vigor and early vigor are important traits associated with seedling stand density (SSD) and weed competitive ability (WCA), which are key factors in direct-seeded rice (DSR) cultivation systems. In this study, we detected a QTL cluster for seed vigor (germination percentage, germination energy, germination index, root length) at a 0.7-Mb interval between RM26797 and RM6680 on Chr.11. The QTL cluster has a significant effect on early vigor under hydroponic culture (root length, total dry weight) and under direct seeding conditions (tiller number, aboveground dry weight). Thus, our combined analysis revealed that the QTL cluster influenced both seed vigor and early vigor. Identifying favorable alleles at this QTL cluster could facilitate the improvement of SSD and WCA, thereby addressing both major factors in DSR cultivation systems.

### Electronic Supplementary Material

Below is the link to the electronic supplementary material.

Supplementary Table S1 Polymorphic markers information used to develop CSSLs.

Supplementary Table S2_Genotype of the CSSL.

Supplementary Fig. S1 Physical linkage map (Mb) of markers in the CSSLs population.

Supplementary Fig. S2 Graphical genotype of the CSSLs.

## Data Availability

All data supporting the conclusions of this article are included in the article and Supplementary files.

## Abbreviations

TPR: Transplanted rice
DSR: Direct-seeded rice
SSD: Seedling stand density
WCA: Weed competitive ability. The others are described in Table 1
