# Supplementary figures and images for: Identification of a Seed Vigor–Related QTL Cluster Associated with Weed Competitive Ability in Direct–Seeded Rice (Oryza Sativa L.)
Source: Rice (N Y). 2023 Oct 13;16:45. doi: 10.1186/s12284-023-00664-x (PMC10575835; doi:10.1186/s12284-023-00664-x)

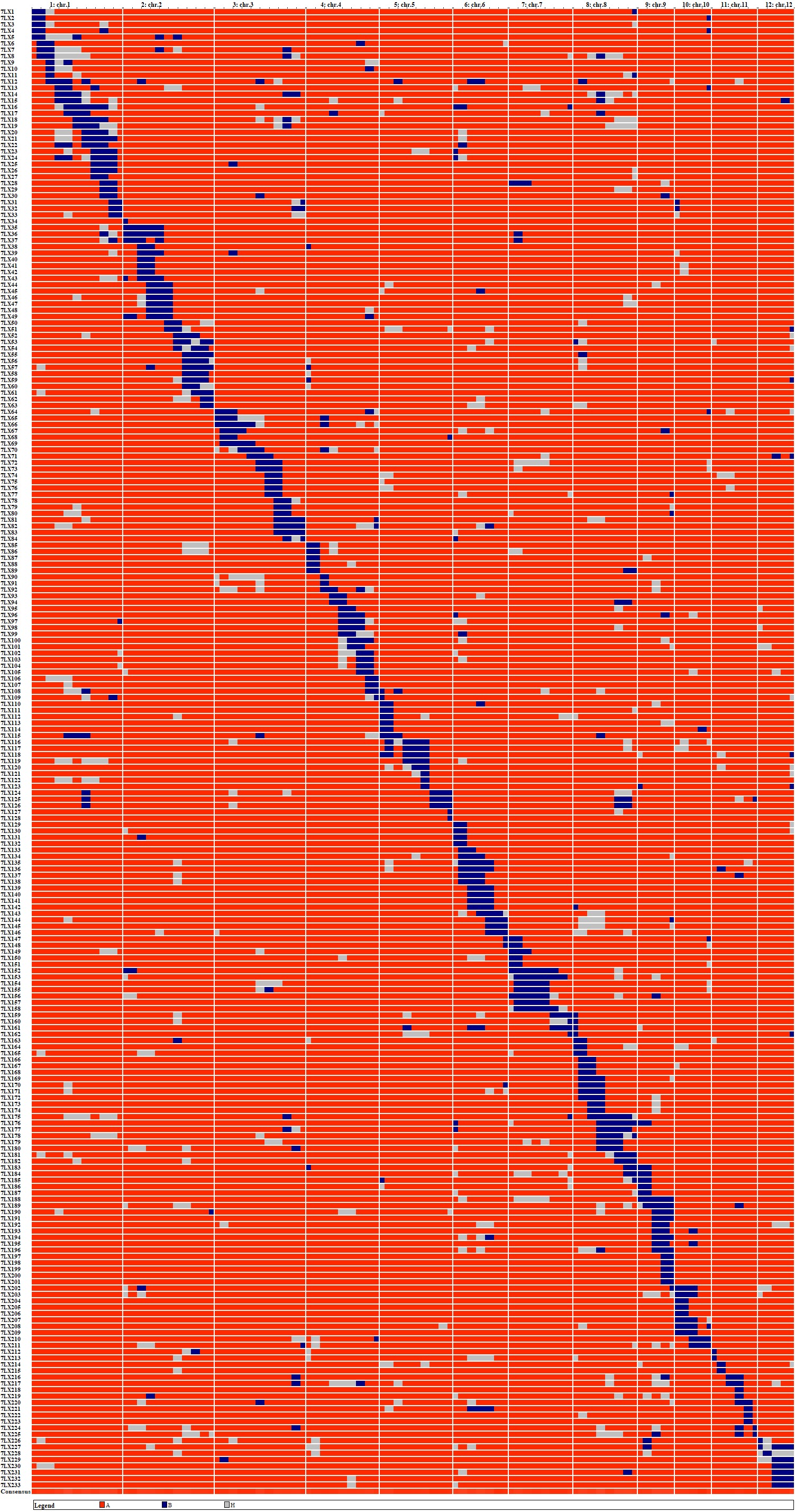

Supplement: Supplementary file 4 — Supplementary Fig. S2 Graphical genotype of the CSSLs. [file 12284_2023_664_MOESM4_ESM.jpg]
